# Implantation of skin-derived precursor Schwann cells improves erectile function in a bilateral cavernous nerve injury rat model

**DOI:** 10.1186/s12610-023-00187-x

**Published:** 2023-05-18

**Authors:** Xiaolei Ma, Wende Yang, Pan Nie, Zhenbin Zhang, Zehong Chen, Hongbo Wei

**Affiliations:** Department of Gastrointestinal Surgery, The Third Affiliated Hospital of Sun Yat-Sen University, Tianhe Road 600, Guangzhou, 510630 China

**Keywords:** Skin-derived precursor, Schwann cell, Erectile dysfunction, Bilateral cavernous nerve injury, Cell therapy, Nerve regeneration, Précurseur dérivé de la Peau, Cellule de Schwann, Dysfonction érectile, Lésion bilatérale du Nerf caverneux, Thérapie cellulaire, Régénération nerveuse

## Abstract

**Background:**

This study was conducted to investigate the therapeutic potential of the skin-derived precursor Schwann cells for the treatment of erectile dysfunction in a rat model of bilateral cavernous nerve injury.

**Results:**

The skin-derived precursor Schwann cells-treatment significantly restored erectile functions, accelerated the recovery of endothelial and smooth muscle tissues in the penis, and promoted nerve repair. The expression of p-Smad2/3 decreased after the treatment, which indicated significantly reduced fibrosis in the corpus cavernosum.

**Conclusions:**

Implantation of skin-derived precursor Schwann cells is an effective therapeutic strategy for treating erectile dysfunction induced by bilateral cavernous nerve injury.

## Résumé

**Contexte:**

Cette étude a été menée pour étudier le potentiel thérapeutique des cellules de Schwann dérivées de la peau pour le traiter la dysfonction érectile survenue dans un modèle de lésion bilatérale du nerf caverneux chez le rat.

**Résultats:**

Le traitement par des cellules de Schwann dérivées de la peau a significativement restauré les fonctions érectiles, accéléré la récupération des tissus endothéliaux et des tissues musculaires lisses du pénis, et a favorisé la réparation nerveuse. L’expression de p-Smad2/3 a diminué après le traitement, ce qui indique une fibrose significativement réduite dans le corps caverneux.

**Conclusion:**

L’implantation de cellules de Schwann dérivées de la peau est une stratégie thérapeutique efficace pour traiter la dysfonction érectile induite par une lésion bilatérale du nerf caverneux.

## Background

Sexual dysfunction is a prevalent postoperative complication of pelvic surgeries. For males, erectile dysfunction (ED) is accepted as the most common sexual dysfunction [1]. ED results from operative damages to the pelvic autonomic nerve (PAN) fibres that restrict fibre-mediated vasodilation of the corpus cavernosum. Thus, PAN-preservation has been widely practiced in the clinic to prevent postoperative ED, and the ED occurrence rate has significantly reduced due to such practice [2, 3]. Despite this, a previous study reported that 47.9% of the patients who received the PAN-preservation treatment still developed ED [4], suggesting that there remains room for improvement. Phosphodiesterase type 5 inhibitors (PDE5Is) have been used as the primary therapeutic option for penile rehabilitation in cases of postoperative ED [5]. However, several large-scale randomised clinical trials have demonstrated that the regular use of PDE5Is following pelvic surgeries cannot prevent persistent deterioration of erectile function [6–8].

The cavernous nerve, projected from pelvic ganglion and reaching the target organ, exerts antagonistic effect on the sympathetic nerve to guarantee the functional vasodilation of the corpus cavernosum. And after it is injuried, the antagonistic effect is weaken, leading to penile cavernosal smooth muscle diastolic disorder and eventually ED [9]. As operative damages to the PAN are the leading cause of postoperative ED, we reasoned that accelerating nerve regeneration may effectively restore such disturbed erectile functions. Endeavors have been made to search for effective and enduring treatments for ED caused by nerve injury. An important method of treating ED induced by nerve injury is transplantation of stem cells. Adipose-derived stem cells have been used to restore the erectile function in rat model in a couple of studies [10, 11]. However, therapeutic effect of adipose-derived stem cell itself did not seem to be satisfying. To obtain the better therapeutic effect, adipose-derived stem cells often need further processing such as transfection with neurotrophic factors, which limited the application. It has been demonstrated that Schwann cells (SCs) can provide a regenerative environment and promote nerve regeneration for periphery nerve injuries [12–14]. However, transplantation of autologous SCs on the sites of peripheral nerve injury has been restricted by SCs’ relatively limited expansion capacity [15, 16] and potential damage to the donor during the harvest. To address this problem, the skin dermis arose as a viable alternative source of SCs, as it contains multipotent progenitor cells that can be successfully differentiated into SCs called skin-derived precursors (SKPs) [17, 18]. Schwann cells originating from SKPs, aslo called skin-derived precursor Schwann cells (SKP-SCs), can express all common markers of SCs, such as p75, S100β, and glial fibrillary acidic protein (GFAP). Furthermore, SKP-SCs have been reported to display a classic bipolar morphology similar to nerve-derived SCs [19] and express a myelinating phenotype when cocultured with axons [20]. Importantly, SKP-SCs could myelinate central nerve system (CNS) axons and repair spinal cord injuries [21, 22], which indicated that SKP-SCs may act as a promising treatment for nerve injury. In this study, we investigated the therapeutic potential of SKP-SCs for the restoration of disturbed erectile functions using a Sprague–Dawley (SD) rat model of bilateral cavernous nerve injury (BCNI).

## Materials and methods

### Cell isolation and characterisation

#### SKP-SCs

SKPs were isolated and cultured as described previously [19, 20]. SKPs were cultured in Dulbecco’s Modified Eagle’s Medium (DMEM)/F-12 supplemented with 20 ng/mL epidermal growth factor (EGF) (Peprotech, 400–25-20), 40 ng/mL fibroblast growth factor (FGF) (Peprotech, 400–29-10), and 2% B27 (Gibco, 17,504,044) at 37 ℃ and 5% CO_2_. SKPs were passaged by mechanically dissociating the spheres and further cultured in a mixture of 75% new medium and 25% conditioned medium. For SC differentiation, the spheres were dissociated and cultured on plates coated with poly-D-lysine and laminin in a SC-differentiation medium composed of DMEM/F-12 with 1% N_2_ (Gibco, 17,502,048) supplement and 10 ng/mL neuregulin-1β (Peprotech, 100–03) for the first 2 weeks. The specified culture medium was then added with 4 μm forskolin (Sigma, F6886) for the subsequent 2 weeks of culture. The secondary passages were collected and frozen at -80 ℃ for further use.

#### Primary SCs

After male SD rats (4 weeks old) were anaesthetised and killed, their bilateral sciatic nerves were removed and transferred to a sterile petri dish. The epineurium and other connective tissues were removed in Hank’s balanced salt solution (HBSS) with 1% penicillin/streptomycin on ice. The nerve bundles were cut into 3 to 5-mm pieces, washed with HBSS, and transferred to a 6-well plate for incubation in DMEM high-glucose medium with 10% fetal bovine serum (FBS) at 37 ℃ and 5% CO_2_. When the cells reached confluence, they were detached and transferred to a 25-cm^2^ culture flask. The SKP-SCs and primary SCs were subjected to immunofluorescence staining for S100β as the marker protein. For further characterisation, SKP-SCs were also stained for myelin basic protein (MBP) and GFAP.

#### Fibroblasts

Skin tissues were obtained from the backs of 4-week-old male SD rats and cut into 5 × 5 mm pieces using sterile scissors. The subcutaneous muscle and adipose tissues were gently removed. The skin tissues were then digested with 0.25% trypsin at 37 ℃ for 30 min, digested in 1% type I collagenase at 37 ℃ for 50 min, passed through a 200-mesh sieve filter, and centrifuged at 1000 rpm at room temperature for 15 min. The supernatant was removed, and the cell pellet was resuspended in Roswell Park Memorial Institute-1640 (RPMI-1640) medium with 10% FBS. The cells were transferred to 25-cm^2^ culture flasks at a density of 1 × 10^4^ cells/mL and cultured at 37 ℃ and 5% CO_2._ The medium was changed every other day. The fibroblasts were subjected to immunofluorescence staining for Vimentin as the marker protein.

### Animal treatment

Male SD rats (10 weeks old weighing 250 to 300 g) were purchased from the Experimental Animal Center of Sun Yat-sen University. These rats were maintained on a 12-h light–dark cycle and provided with access to water ad libitum at the Center for Experimental Animals of the South China Agriculture University. The study protocol was reviewed and approved by the Institutional Animal Care and Use Subcommittee of the South China Agriculture University. Twenty SD rats were randomly divided into the following four groups (n = 5): sham group, and three BCNI groups. Rats in the BCNI groups were treated with phosphate buffered phosphate-buffered saline (PBS), SKP-SCs, and primary SCs around the major pelvic ganglia (MPG).

For the BCNI model, rats were first weighed and anaesthetised with 2.5 to 3% isoflurane. The nerve crush injury was induced at a site 2 to 5 mm distal to the MPG as previously described [23]. The sham group was subjected to the same surgical procedures without nerve-crushing and cell-implantation. The implantation of SKP-SCs and primary SCs were performed as previously described [24]. Fibrin scaffolds of the cells in PBS were prepared with a Porcine Fibrin Sealant Kit (Hangzhou Puji Medical Technology Development Co. Ltd., Hangzhou, China) according to the manufacturer’s instructions. A mixture of cells and fibrin scaffolds was implanted around the MPG. For the SKP-SCs and SCs groups, around 1 × 10^6^ cells in 100 µL cell-fibrin scaffolds were implanted per rat. For the PBS group, 100 µL of PBS-fibrin scaffolds was treated per rat.

### Preparation of conditioned medium and enzyme-linked immunosorbent assay

Three types of cells (SKP-SCs, primary SCs, and fibroblasts) were cultured in 6-well plates. When the confluence reached 90%, the medium was substituted with 1 mL serum-free medium, and the cells were incubated for an additional 48 h. The supernatant was collected after centrifugation at 3000 rpm at 4 ℃ for 20 min and stored at -80 ℃ for enzyme-linked immunosorbent assay **(**ELISA). The levels of glial cell-derived neurotrophic factor (GDNF) (Meimian, MM-0201R1), brain-derived neurotrophic factor (BDNF) (Meimian, MM-0209R1), monocyte chemotactic protein 1 (MCP-1) (Meimian, MM-0099R1), and Collagen VI (Col VI) (Meimian, MM-50256R1) were quantified in the conditioned media of the specified three cell types.

### Chemotaxis of M0 macrophages

M0 macrophages were differentiated from THP-1 (Procell, CL-0233) cells as previously described [25]. Briefly, THP-1 cells were incubated with 320 nM Phorbol 12-myristate 13-acetate (PMA) for 48 h and differentiated into M0 macrophages. For the confirmation of macrophage subtypes, CD11b was used as the marker for M0 macrophages. The stained cells were analysed using a flow cytometer to determine the percentages of positively-stained cells using the BD FACSDiva software. The M0 macrophages were then subjected to cell sorting using BD FACSAriaIII. Here, CD11b^+^ cells were collected and cultured for the chemotaxis assay. The upper chambers of an 8-μm membrane-based culture insert (PIEP12R48, Millipore) were filled with serum-starved primary M0 macrophages (1 × 10^5^ cells in 300 μL per well), and the lower chambers were filled with the conditioned media of different cell types or serum-free medium. After incubating at 37 ℃ and 5% CO_2_ for 24 h, the cells remaining on the upper surface of the membrane were first gently wiped off with a cotton swab. The cells remaining on the membrane were then fixed with 4% paraformaldehyde and stained with 0.1% crystal violet. The cells that migrated to the lower surface were analysed using a microscope.

### Neuritogenesis in PC12 cells

To evaluate the neurotrophic growth functions of SKP-SCs and primary SCs, PC12 cells were separately cocultured with either SKP-SCs or primary SCs. The percentage of neurite outgrowth and the number of neurites originating from the soma were then observed [26]. The lower chambers of an 8-μm membrane-based culture insert (PIEP12R48, Millipore) contained PC12 cells that were pre-treated with 50 ng/μL β-nerve growth factor (Peprotech, 450–01-20) for 24 h to stop their proliferation and induce neurite outgrowth [26]. The upper chambers were filled with SKP-SCs or primary SCs (1 × 10^6^ cells in 300 μL per well) in a serum-free medium. After co-culturing for 48 h, neuritogenesis was measured as previously described [27]. Briefly, neurite length was measured by the ratio of neurite length to the size of soma, and the measurements were defined as follows. ‘L0’ for cells with no neurites, ‘L1’ for cells whose neurite length was shorter than the size of the soma, ‘L2’ for cells with neurite length between the original size of the soma and twice the size of the soma, and ‘L3’ for cells whose neurite length was longer than twice the size of the soma.

### Evaluation of erectile functions

The erectile functions of the subjects were evaluated 2 weeks after the treatments, in which the mean arterial blood pressure (MAP) and intracavernous pressure (ICP) of the subjects were recorded continuously as previously described [28]. Under deep anaesthesia, a midline incision was made from the neck to the upper thorax to expose the right carotid artery. Next, a heparinised 24-gauge Silastic cannula was fixed on the surgery site to measure the MAP. The skin of the penis was stripped off to insert a heparinised 25-gauge butterfly needle for ICP measurements. The cannula was connected to a BL-420S Biological Functional System (Chengdu Taimeng Technology Ltd., Chengdu, China) for continuous assessment and recording of ICP. The MPG and cavernous nerve were exposed via a midline incision and stimulated by a bipolar electrode. The stimulus parameters were set at 1.5 mA, 20 Hz, 0.2 ms pulse width, and 50 s duration [28]. The erectile functions were assessed by the ratios of maximum ICP (mICP) to MAP. The MPG and penis were harvested for histological analysis.

### Masson’s trichrome staining

A portion of the penis was cut and washed with PBS, then immediately fixed with 4% paraformaldehyde. The paraffin-embedded penis was cut into 5-µm-thick slices for Masson’s trichrome staining. As described previously, the staining results were used to determine the collagen-to-smooth muscle ratio in the corpus cavernosum [28]. The smooth muscle cells within the corpus cavernosum appeared red, and collagen fibrils appeared blue. The stained sections were photographed using a camera, analysed using the Image-Pro Plus 6.0 software (Media Cybernetics, Rockville, MD, USA).

### PHK26 for cell labeling

PHK26 kit (Sigma-Aldrich) was used for cell labeling following the manufacturer’s instructions. Briefly, the cells were digested and washed with a serum-free medium for three times. Next, the cells were suspended in Diluent C solution to which PKH26 ethanolic dye solution was added. The mixture was gently mixed for dispersion. The cell-suspension was then incubated for five minutes at room temperature. An equal volume of FBS-supplemented medium was added to the suspension to stop the process. The cells were centrifuged, washed, and resuspended in a complete medium. The PHK26-labeled cells were transplanted around the MPGs of BCNI rats and imaged by a laser scanning confocal microscope on days 1, 3, and 7.

### Immunofluorescence staining

The remaining sections of the penis and MPG were collected and prepared as 10-μm frozen slices. They were stored at -30 ℃ for subsequent use. Methyl alcohol was used to fix the slices. The slices were then washed with PBS and blocked with a mixture of bovine serum albumin and Triton X-100. For penis slices, antibodies against neuronal nitric oxide synthase (nNOS) (Abcam, 1:200, ab1376), endothelial nitric oxide synthase (eNOS) (Cell Signaling Technology, 1:100, #32,027), and desmin (Abcam, 1:100, ab32362) were applied. For MPG slices, a nerve growth factor (NGF) (Abcam, 1:200, ab52918) antibody was applied. DyLight 488- and 556-conjugated antibodies were used as secondary antibodies (Invitrogen, 1:500). The nuclei were stained with DAPI (0.5 μg/mL, Invitrogen). A confocal laser scanning microscope (Zeiss LSM 710) was used to observe and image the stained slices.

### immunohistochemical staining

The penile tissues were harvested and fixed with 4% paraformaldehyde. Next, they were embedded in paraffin and sectioned (5 μm) for immunohistochemical staining. Methanol with 0.3% H_2_O_2_ was added to the tissues to inactivate the endogenous peroxidases. The recovery of antigen was performed with 0.1% trypsin for 30 min at 37 ℃, followed by a couple of rinses with PBS. After blocking with 1% BSA for 1 h at room temperature, the sections were incubated with primary antibodies against p-Smad2/3 (Affinity, 1:100, AF3367) and transforming growth factor-β1 (TGF-β1) (Servicebio, 1:400, GB11179). The sections were then rinsed, incubated with horseradish peroxidase for 1 h, and rinsed twice with PBS. The nuclei of all sections were counterstained with haematoxylin. The sections were placed on coverslips and examined with a microscope. The results were expressed by average optical density (AOD) measured with Image J 1.53 k (National Institutes of Health).

### Statistical analysis

The results were analysed using GraphPad Prism 5 (GraphPad Software, La Jolla, CA, USA). The data were presented as mean ± standard deviation. The student’s unpaired t-test was used for two-group comparisons. Multiple-group comparisons were carried out by one-way ANOVA followed by the S–N-K test using the SPSS 16.0 software (SPSS Inc.). *P* < 0.05 was considered statistically significant; * for *p* < 0.05, ** for *p* < 0.01, and *** for *p* < 0.001.

**Fig. 1:**
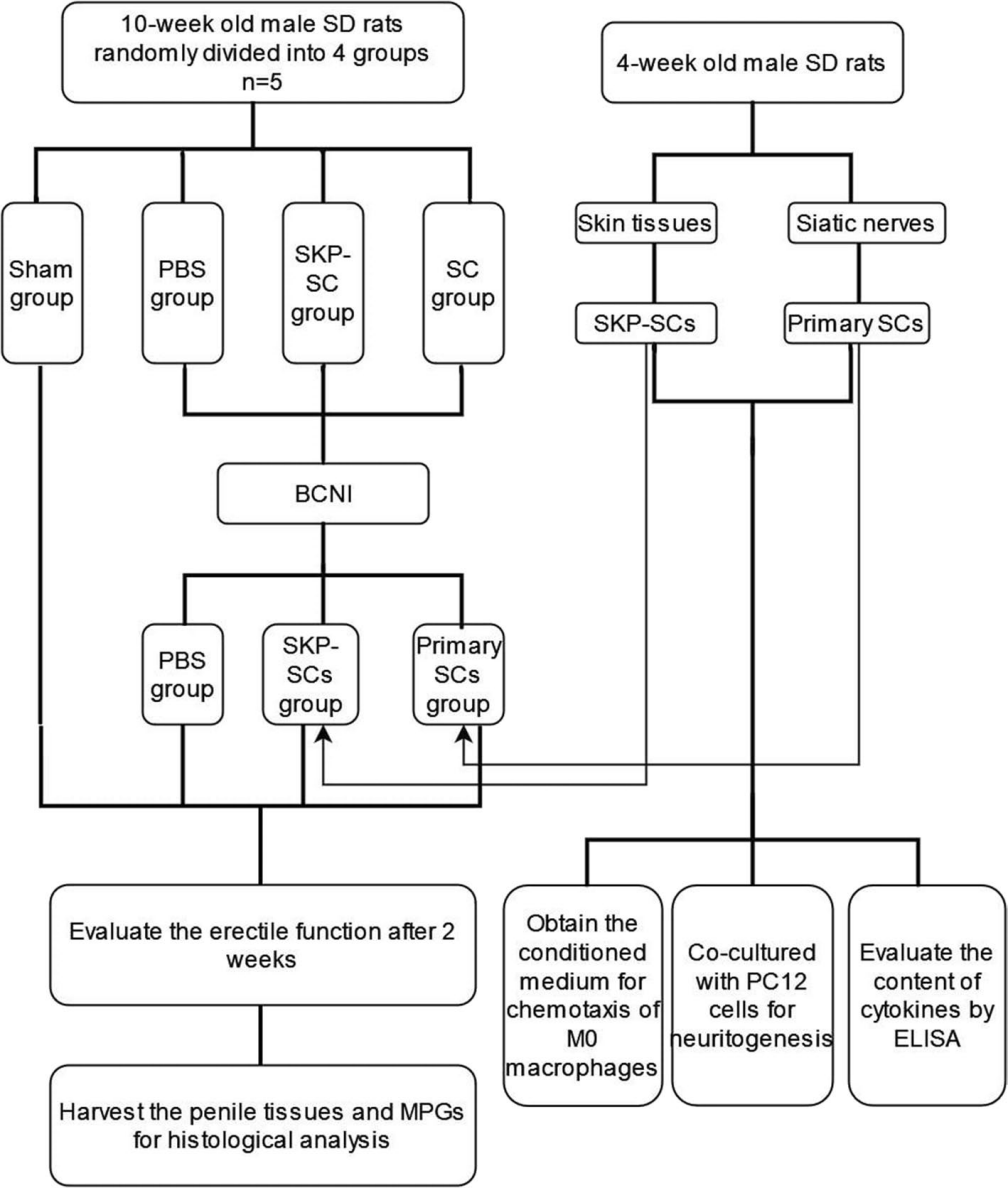
Flowchart of this study

The design idea of this study is shown in Fig. 1.

## Results

### Isolation and characterisation of SKP-SCs, primary SCs, and fibroblasts

**Fig. 2:**
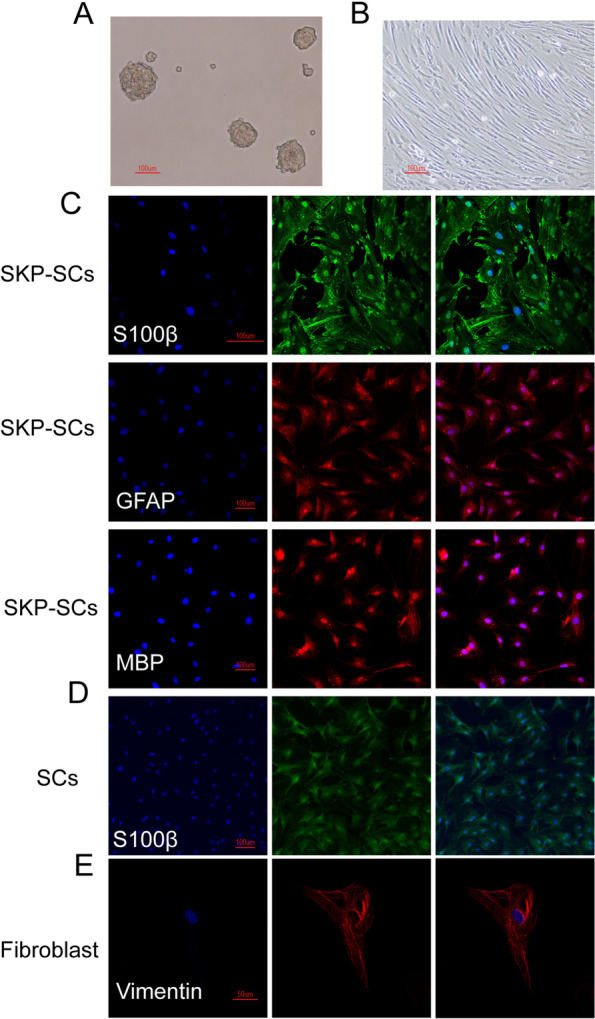
Morphology of skin-derived precursors, skin-derived precursors Schwann cells, primary Schwann cells and fibroblasts. **A** SKPs grew as spheres after 1 week’s incubation. **B** After 2 weeks of differentiation in Schwann cell medium, cells presented a bipolar morphology of Schwann cells and arranged in parallel arrays. **C** SKP-SCs were stained for SC marker S100β(green), GFAP(red) and MBP(red). Nuclei were stained for DAPI(blue). **D** Primary SCs were stained for SC marker S100β(green). Nuclei were stained for DAPI(blue). **E** Fibroblasts were stained for fibroblasts marker Vimentin(red). Nuclei were stained for DAPI(blue). SKPs: skin-derived precursors; SKP-SCs: skin-derived precursors Schwann cells; GFAP: glial fibrillary acidic protein; MBP: myelin basic protein; SCs: Schwann cells

SKPs were isolated from the skin tissues of 3-week-old SD rats and cultured in vitro. These SKPs initially displayed a typical sphere morphology (Fig. 2A). After a 2-week culture in a SKP-SC differentiation medium, SKPs showed a typical bipolar morphology of SCs arranged in parallel arrays (Fig. 2B). To characterise the SKP-SCs, we evaluated their expressions of S100β, GFAP, and MBP using fluorescence microscopy. The results showed that most cells expressed green fluorescent protein, indicating that the SKPs were successfully differentiated into SCs (Fig. 2C). We also examined the expression of S100β in primary SCs. The immunofluorescence staining showed that most cells expressed S100β, thereby verifying the cell type (Fig. 2D). Furthermore, the positive immunofluorescence staining of Vimentin indicated the successful extraction and culture of fibroblasts (Fig. 2E).

### Effects of SKP-SCs on improving erectile function in BCNI model rats

**Fig. 3:**
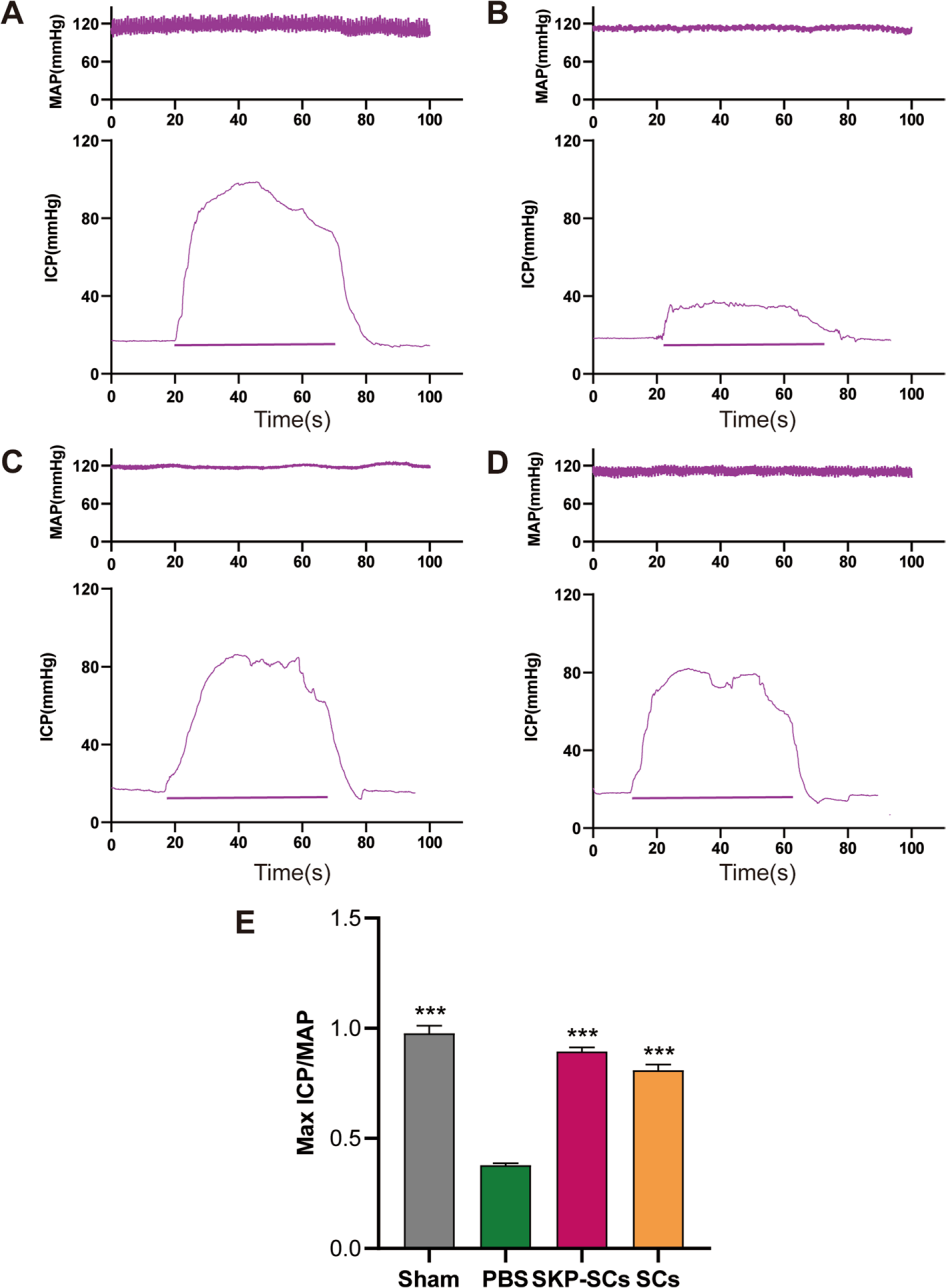
Skin-derived precursors Schwann cells had an effect on restoring erectile function of bilateral cavernous nerve injury rats. **A**-**D** Representative ICP of sham group, BCNI rats treated with PBS, SKP-SCs and primary SCs respectively. The black bars means the 50 s of electrical stimulation. **E** The ratio of maximal ICP to MAP. The values depict as the mean ± standard deviation from 5 animals per group. ***p < 0.001; the student’s unpaired t-test was used for two-group comparisons; multiple-group comparisons were carried out by one-way ANOVA followed by the S–N-K test. ICP: intracavernous pressure; BCNI: bilateral cavernous nerve injury; PBS: phosphate buffered saline; SKP-SCs: skin-derived precursors Schwann cells; SCs: Schwann cells; MAP: mean arterial blood pressure

We implanted SKP-SCs and primary SCs around the MPG after BCNI was induced in rat subjects. After stimulation of cavernous nerve, MAP and ICP were recorded 2 weeks after the treatments. The ratio of ICP to MAP reflected penile erectile function. The mICP-to-MAP ratios were within the normal range. As expected, the BCNI group showed a significant decline in the mICP-to-MAP ratio, suggesting that the subjects’ erectile functions were severely impaired due to the injury. The implantation of SKP-SCs and primary SCs significantly improved the mICP-to-MAP ratios compared with that of the PBS-treated group. Both SKP-SCs and primary SCs considerably recovered the erectile functions of the subjects 2 weeks after the treatments. The erectile function of the SKP-SC group was not significantly different from that of the primary SC group (Fig. 3A-E).

### Effects of SKP-SCs on protecting the smooth muscle content and preventing fibrosis in the corpus cavernosum

**Fig. 4:**
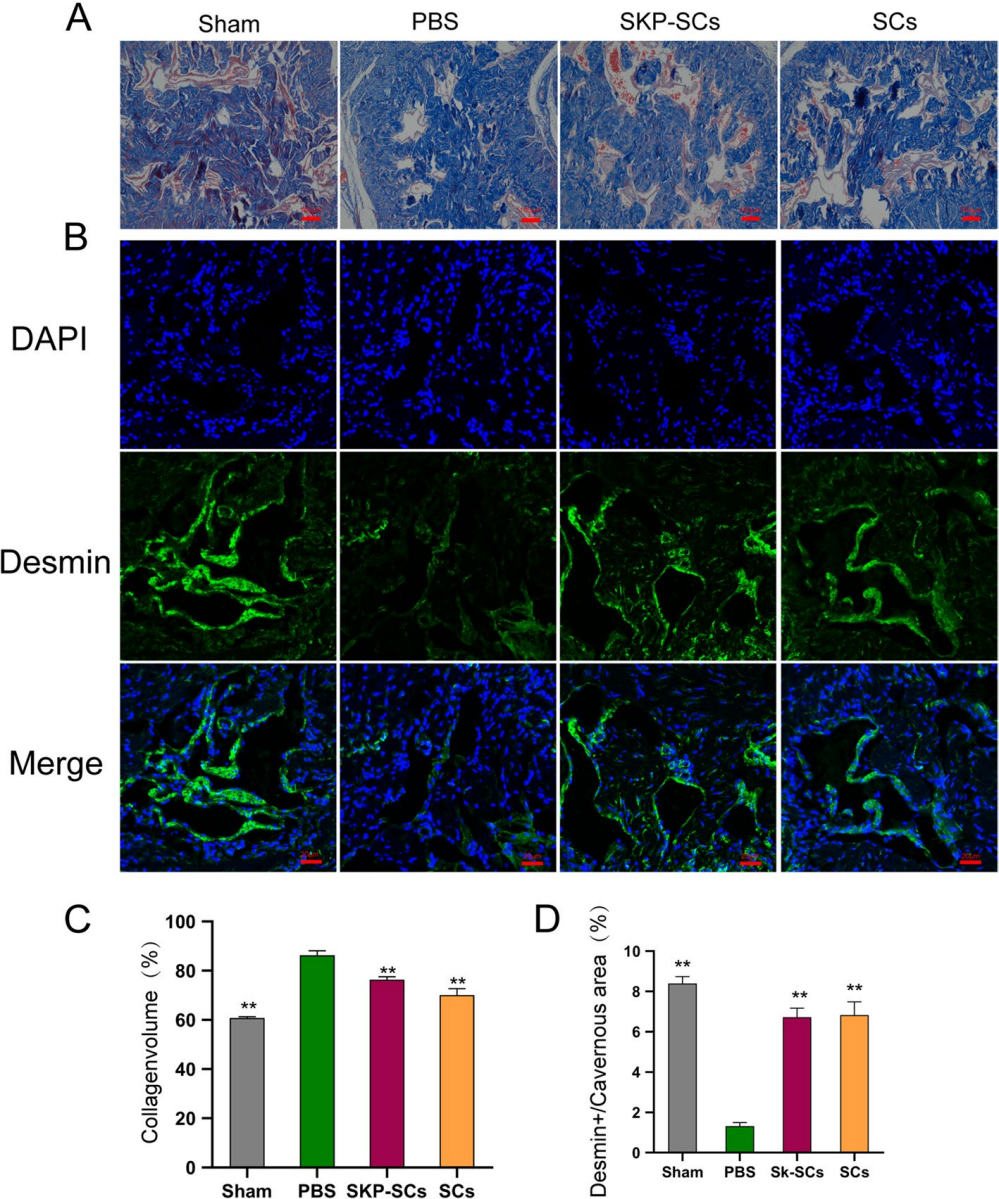
Skin-derived precursors Schwann cells protected the smooth muscle content and prevented the fibrosis process in corpus cavernous. **A** Masson trichrome staining of penile cross section tissues specimen of each group. Red and blue represented muscle and collagen content respectively. **B** Immunofluorescence staining for desmin(green) in penile cross section tissues specimen. **C** Quantitative analysis of Masson trichrome staining results. The ratio of collagen to smooth muscle showed the degree of fibrosis. **D** Quantitative analysis of the desmin immunofluorescence positive area. All the data were depicted as mean ± standard deviation from 5 animals per group. **p < 0.01; the student’s unpaired t-test was used for two-group comparisons; multiple-group comparisons were carried out by one-way ANOVA followed by the S–N-K test

BCNI can cause smooth muscle atrophy and fibrosis in the corpus cavernosum fibrosis. The collagen-to-smooth muscle ratio is a marker of fibrosis in the corpus cavernosum. We found that for the BCNI group, the collagen-to-smooth muscle ratio significantly increased, indicating that severe fibrosis had occurred 2 weeks after the injury was induced. Compared with the PBS group, the SKP-SC and primary SC groups showed significantly lower collagen-to-smooth muscle ratios. There were no significant differences between the SKP-SC and primary SC groups (Fig. 4A, C). We also analyzed the expression of desmin by immunofluorescence staining to evaluate further the changes in the smooth muscle content upon described treatments. The results showed that the BCNI group showed a significantly lower level of desmin than the sham group, indicating that the injury had indeed caused atrophy of the smooth muscles in the corpus cavernosum. The expressions of desmin in the SKP-SC and primary SC groups were significantly higher than that in the PBS group. Again, there were no significant differences between the SKP-SC and primary SC groups in terms of desmin expression (Fig. 4B, D).

### Effects of SKP-SCs on promoting nerve regeneration

**Fig. 5:**
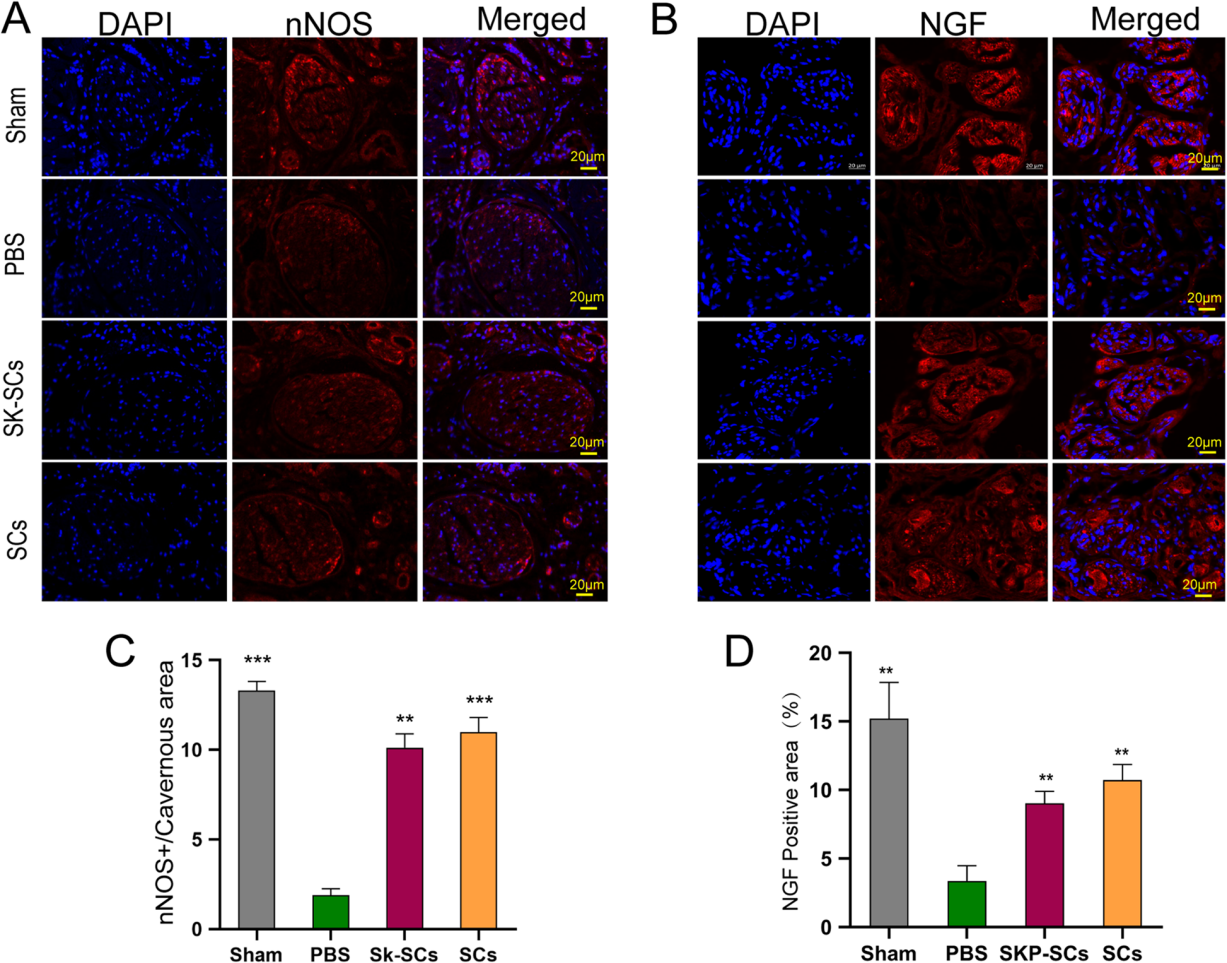
Skin-derived precursors Schwann cells promoted the nerve regeneration in vivo. **A** Immunofluorescence staining for nNOS in cross section of penis tissues specimen. And nNOS positive neural fibers were stained red. **B** Immunofluorescence staining for NGF in MPG tissues section. The NGF positive areas were stained red. **C**, **D** Quantitative results of immunofluorescence results for nNOS and NGF, respectively. All the data were depicted as mean ± standard deviation from 5 animals per group. **p < 0.01, ***p < 0.001; the student’s unpaired t-test was used for two-group comparisons; multiple-group comparisons were carried out by one-way ANOVA followed by the S–N-K test. nNOS: neuronal nitric oxide synthase; NGF: nerve growth factor; MPG: major pelvic ganglion

**Fig. 6:**
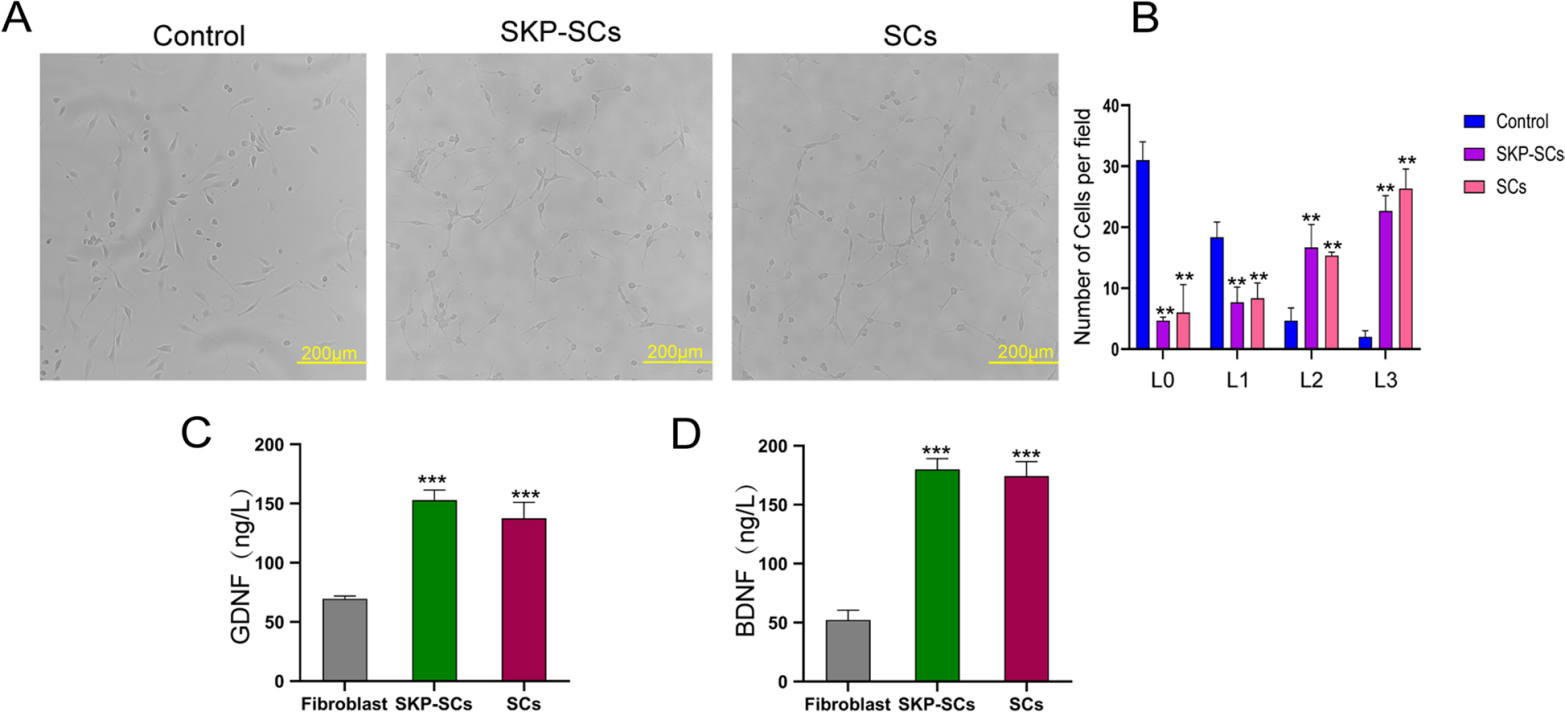
Skin-derived precursors Schwann cells promoted the nerve regeneration in PC12 cells. **A** Neurites outgrowth of PC12 cells. **B** Neurites length of PC12 cells treated with different conditions. **C**, **D** ELISA assay of GDNF and BDNF levels of different conditioned medium. All values are represented as the mean ± standard deviation from three independent experiments. **p < 0.01, ***p < 0.001; the student’s unpaired t-test was used for two-group comparisons; multiple-group comparisons were carried out by one-way ANOVA followed by the S–N-K test. ELISA: enzyme-linked immunosorbent assay; GDNF: glial cell-derived neurotrophic factor; BDNF: brain-derived neurotrophic factor

We evaluated the expression of nNOS in the corpus cavernosum 2 weeks after the implantation of SKP-SCs and primary SCs. The results of immunofluorescence analysis indicated that the expressions of nNOS in the SKP-SC and primary SC groups were significantly higher than that in the PBS group and lower than that in the sham group. Collectively, these results indicated that the implantation of SKP-SCs could significantly promote nerve regeneration (Fig. 5A, C). We also evaluated the expressions of NGF in MPG tissues by immunofluorescence staining. The NGF expressions in the SKP-SC and primary SC groups were significantly higher than that in the PBS group. These results indicated that the implantation of SKP-SCs and primary SCs promoted the process of nerve regeneration (Fig. 5B, D) as expected. The expression of nNOS did not significantly differ between the SKP-SC and primary SC groups. After 48 h of coculture, neurite lengths were recorded and the effects of different treatments were compared. The results indicated that the numbers of L0 and L1 cells were significantly higher in the control group than in the SKP-SC and primary SC groups. However, the numbers of L2 and L3 cells were significantly higher in the SKP-SC and primary SC groups than in the control group. There was no significant difference between the SKP-SC and primary SC groups (Fig. 6A, B) in terms of neurite lengths. The levels of two types of neurotrophic factors, GDNF and BDNF, in the conditioned media of SKP-SCs and primary SCs were analysed by ELISA. The results showed that the levels of GDNF and BDNF were significantly higher in SKP-SC and primary SC groups than those of fibroblasts (Fig. 6C, D).

### Effects of SKP-SCs on increasing endothelial tissue content in the corpus cavernosum

**Fig. 7:**
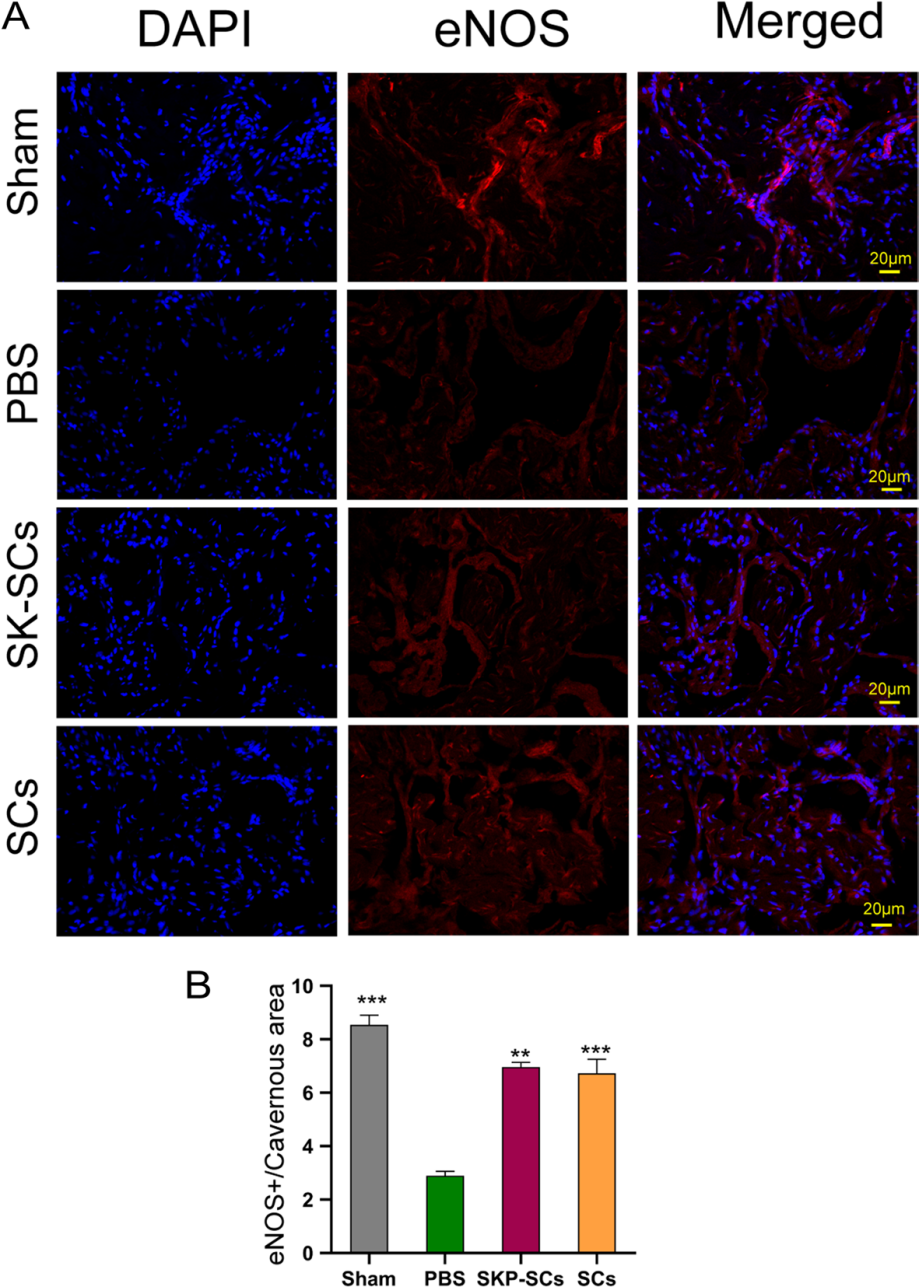
Skin-derived precursors Schwann cells preserved the endothelial content of corpus cavernosum. **A** Immunofluorescence staining for eNOS (red) in penile cross section tissues specimen. **B** Quantitative analysis of the eNOS immunofluorescence positive area. All the data were depicted as mean ± standard deviation from 5 animals per group. **p < 0.01, ***p < 0.001; the student’s unpaired t-test was used for two-group comparisons; multiple-group comparisons were carried out by one-way ANOVA followed by the S–N-K test. eNOS: endothelial nitric oxide synthase

Functional and integrated endothelial tissues in the corpus cavernosum are essential for erection. We performed immunofluorescence staining to evaluate the expression of eNOS, which reflects the integrity of the functional endothelium. The results showed that the expressions of eNOS in the SKP-SC and primary SC groups were significantly higher than that in the PBS group. The SKP-SC and primary SC groups did not show significant differences in eNOS expression. The results demonstrated that the implantation of SKP-SCs and primary SCs could increase the endothelial tissue content (Fig. 7A, B).

### *Effects of SKP-SCs on enhancing the chemotaxis of M0 *in vitro

**Fig. 8:**
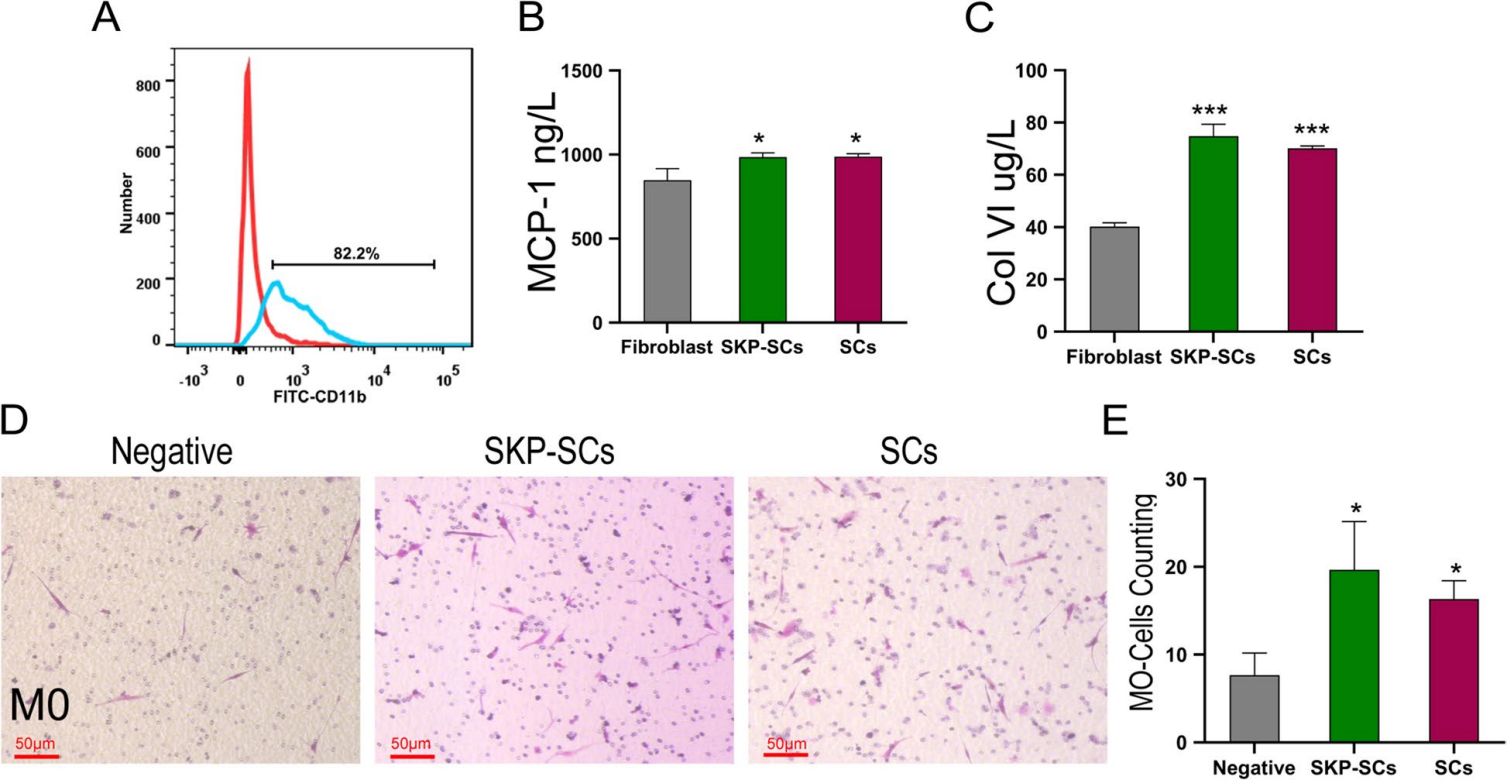
Skin-derived precursors Schwann cells enhanced chemotaxis of M0 macrophages and secreted chemokines. **A** Induction and identification of M0 macrophages. Flow cytometry showed M0 macrophages expressed more CD11b after differentiated from THP-1 cells. **B**, **C** ELISA assay of MCP-1 and Col VI levels of different conditioned medium. **D** M0 macrophages chemotaxis under the condition of different conditioned medium visualized by crystal violet. **E** Qualification of cells that migrated. All values are represented as the mean ± standard deviation from three independent experiments, each with three replicates. *p < 0.05, ***p < 0.001; the student’s unpaired t-test was used for two-group comparisons; multiple-group comparisons were carried out by one-way ANOVA followed by the S–N-K test. ELISA: enzyme-linked immunosorbent assay; MCP-1: monocyte chemotactic protein 1; Col VI: Collagen VI

To quantify M0 macrophages that underwent chemotaxis, we analyzed their surface marker, CD11b, using flow cytometry. The results showed 82.2% positivity for CD11b after cell sorting (Fig. 8A). When injuries occur, macrophages assemble at the sites of injury to clear the harmful debris and promote repair. The results indicated that a significantly elevated level of M0 macrophage chemotaxis was induced in the SKP-SC and primary SC groups than in the control group. We did not observe any significant differences between the SKP-SC and primary SC groups (Fig. 8D, E) in M0 macrophage chemotaxis. To further investigate the mechanism of SKP-SC-promoted chemotaxis of M0 macrophages, we used ELISA to quantify MCP-1 and Col VI levels in the conditioned media. The results showed that the level of MCP-1 was significantly higher in the conditioned media of SKP-SCs and primary SCs than that in the conditioned medium of fibroblasts. As for Col VI, the differences remained significant. The level of MCP-1 was significantly higher in the conditioned media of SKP-SCs and primary SCs than that in the conditioned medium of fibroblasts (Fig. 8B, C).

### Effects of SKP-SCs on preventing BCNI-induced fibrosis in the corpus cavernosum by downregulating the TGF-β/Smad2/3 pathway

**Fig. 9:**
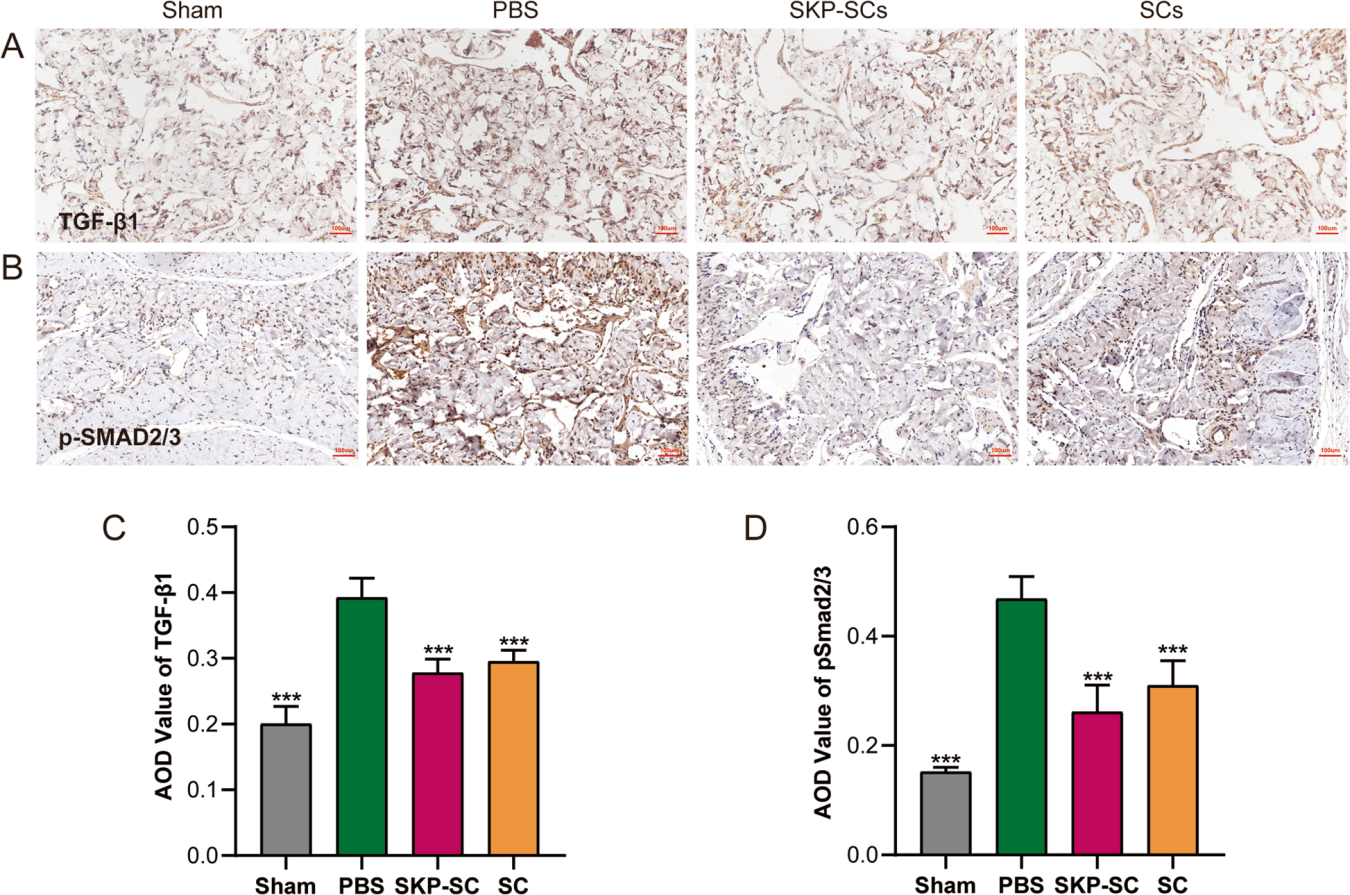
Skin-derived precursors Schwann cells prevented the fibrosis process in corpus cavernosum by downregulating the transforming growth factor/Smad2/3 pathway. **A** Immunohistochemical staining for TGF-β1 (brown) in penile cross section tissues specimen. **B** Immunohistochemical staining for p-Smad2/3 (brown) in penile cross section tissues specimen. **C**, **D** Quantitative analysis of the AOD value of the TGF-β1 and p-Smad2/3. All the data were depicted as mean ± standard deviation from 5 animals per group. ***p < 0.001; the student’s unpaired t-test was used for two-group comparisons; multiple-group comparisons were carried out by one-way ANOVA followed by the S–N-K test. TGF-β1: transforming growth factor-β1; AOD: Average optical density

In response to tissue damage, TGF-β1 stimulates the phosphorylation of Smad2/3, which is activated by serine/threonine phosphorylation and binds to the co-activator to form a complex. This complex subsequently moves into the nucleus and regulates the transcription of fibrosis-related target genes. We used immunohistochemical staining to quantify the changes in the expression of TGF-β1 and p-Smad2/3 upon described treatments. The PBS groups showed the highest levels of TGF-β1 and p-Smad2/3, indicating that severe fibrosis had occurred in the corpus cavernosum 2 weeks after BCNI. For the SKP-SC and primary SC groups, the TGF-β1 and p-Smad2/3 levels were significantly lower than those in the PBS group. Analysis of average optical density (AOD) values of the immunohistochemically stained section revealed that the TGF-β1 and p-Smad2/3 levels were significantly higher in the PBS group than those in the other groups (Fig. 9A, B, C, and D).

### In vivo* time course of SKP-SCs and primary SCs in BCNI rats*

**Fig. 10:**
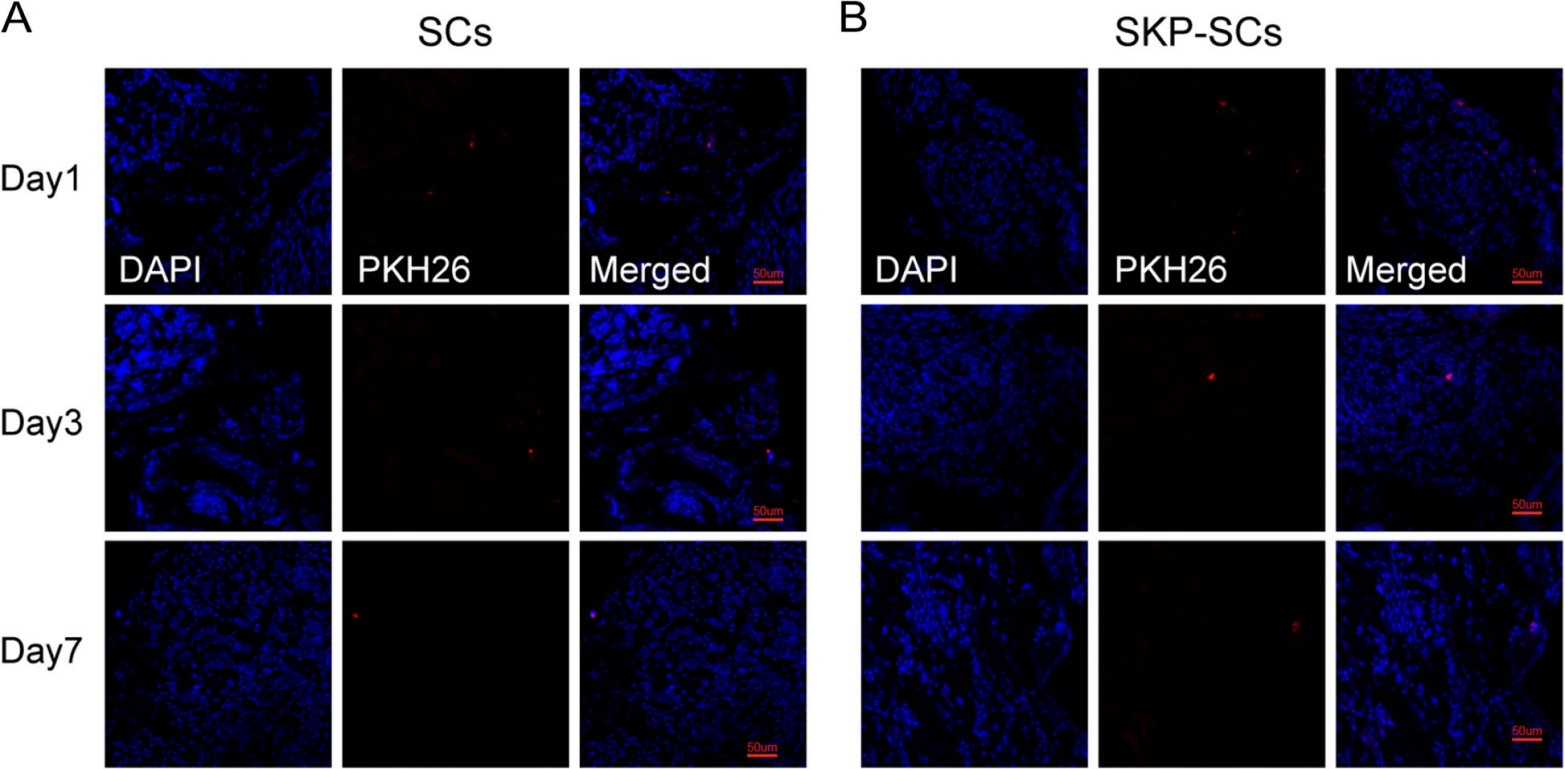
Analysis of skin-derived precursors Schwann cells’ and primary Schwann cells’ survival over time in vivo. **A** The PKH26-labled primary SCs were detectable in or around MPG tissues 1 day, 3 days and 7 days after the transplantation of the cells around the MPG. **B** The PKH26-labled SKP-SCs were also detectable at the 3 time points. SKP-SC: skin-derived precursor Schwann cell; MPG: major pelvic ganglion; SCs: Schwann cells

To evaluate the in vivo survival of the transplanted SKP-SCs and primary SCs over time, we stained these cells with the PKH26 dye. The PKH26-stained cells were analyzed on days 1, 3, and 7 after being transplanted around the MPG of the rat subjects. The fluorescence of both primary SCs and SKP-SCs could be detected in and around the MPG tissues throughout the 7-day monitoring, indicating that primary SCs and SKP-SCs could survive in vivo for at least seven days to exert their nerve regeneration functions (Fig. 10A and B).

## Discussion

The application of primary SCs for treating peripheral nerve injury has been proved effective so far. However, invasive procedures to obtain primary SCs lead to inevitable dysfunctions. Moreover, the expansion of adult SC cultures requires the presence of serum, and this may promote the undesired proliferation of contaminating fibroblasts [29, 30]. Thus, a viable alternative source for primary SCs is currently needed for effective ED therapy. SKPs are self-renewing and multipotent precursor cells isolated from the dermis of either human or rodent skin [17]. The gene expression profile of SKPs is similar to that of embryonic neural crest stem cells. SKPs exhibit a strong potential for differentiation to SCs and neurons of the peripheral nerves [31]. In an appropriately-conditioned environment, specific cytokines can induce the SKPs to differentiate into SCs [19, 20]. Moreover, these SKP-SCs can express a myelinating phenotype when cocultured with injured peripheral and CNS cells both in vivo and in vitro [22, 32]. SKP-SCs can also express a repairing phenotype by generating a battery of cytokines, including IFN-γ, IL1-β, and IL-6. Applying SCs could significantly improve the functions of injured peripheral and CNS nerves, such as sciatic nerves and the spinal cord [22, 32]. However, the approach of using SKP-SCs to repair erectile dysfunctions caused by nerve injury has not been explored yet. In this study, we implanted SKP-SCs around the MPG of SD rats with BCNI to investigate the therapeutic potential of SKP-SCs for treating erectile dysfunctions. Two weeks after the surgery and appropriate treatments, the analyses of the subjects' erectile functions and their corpus cavernosum morphology indicated that SKP-SCs could repair erectile dysfunction significantly better than the control group. The leading causes of nerve injury-induced ED include fibrosis and apoptosis in the corpus cavernosum, which cause irreversible damages in most cases [33]. As such, the conventional PED5I therapy has yielded mediocre responses in patients with nerve injury-induced ED [34]. In this respect, we explored the use of SKP-SCs to repair the damaged cavernous nerve and MPG in the early stage of the injury to prevent fibrosis and apoptosis in the corpus cavernosum.

The paracrine release of cytokines by the transplanted SKP-SCs is considered one of the most important mechanisms for the regeneration of damaged nerves. Here, GDNF alone or combined with BDNF can trigger the migration of SCs and increase the survival rate of neurons [35]. Our study demonstrated that SKP-SCs could secrete sufficient levels of BDNF and GDNF needed for the enrichment of cytokines at the injury sites. To further evaluate the nerve-regenerating ability of SKP-SCs, we cocultured them with PC12 cells to study the effects on neuritogenesis. The results indicated that SKP-SCs could effectively increase both the length and number of neurites in PC12 cells.

Besides the paracrine release of cytokines that can contribute to neurite growth, it has been demonstrated that SCs can also recruit macrophages for nerve repair [36]. Upon damage to the peripheral nerve, the units of axon/SC disintegrate in which SCs release various chemokines and cytokines, including MCP-1, IL-1α, and IL-1β, in a paracrine manner to chemoattract macrophages to the injury site [37–39]. SCs can also produce Col VI at the injury site, which can induce the polarisation of macrophages from the M0 to M2 subtype [40]. For the chemoattraction assay, we used the supernatants of SKP-SCs and primary SCs to chemoattract M0 macrophages. The results revealed that SKP-SCs could exert a significant level of macrophage chemoattraction comparable to that of primary SCs. The M2 macrophage subtype shows excellent therapeutic potential, given its anti-inflammatory effects and ability to boost nerve tissue regeneration [41]. We rationally reasoned that SKP-SCs could attract M0 macrophages to the injury site and induce their polarisation from M0 to M2 subtype to exert the functions of nerve regeneration. The expression of NGF in the MPG was evaluated to study the effects of SKP-SCs on nerve regeneration. The NGF expression in the SKP-SC group was significantly higher than that in the PBS group. This result indicated that SKP-SCs could effectively repair the injured MPG and cavernous nerve to a degree comparable to that of SCs [42].

It is well known that normal penile erectile functions require the characteristic nitrogen nerves, intact endothelium, and functional penile smooth muscle to work in concert [43]. When MPG or CN is subjected to denervation, changes in the corpus cavernosum lead to irreversible erectile dysfunction. Here, nNOS-positive neurons represent penile projection neurons critical to penile erection [44]. Therefore, we used immunofluorescence staining to evaluate the expression of nNOS in the dorsal nerves of the penis. The expressions of nNOS in the primary SC and SKP-SC groups were similar and significantly higher than that in the PBS group. Therefore, it was concluded that the implantation of SKP-SCs could effectively protect nNOS-positive neurons for the restoration of erectile functions. eNOS is specific to endothelial cells in the corpus cavernosum, and thus, its expression level can reflect the integrity of endothelial cells. As aforementioned, the integrity of endothelial cells is essential for normal erectile functions. We, therefore, evaluated the expression of e-NOS in the corpus cavernosum by immunofluorescence staining. The results showed that both the SC and SKP-SC groups showed significantly enhanced and similar expressions of eNOS compared with that of the control group, indicating that transplantation of SKP-SCs could effectively protect the integrity of endothelial cells.

Fibrosis could be observed in the corpus cavernosum as early as one week after the injury in CN or MPG [45]. After BCNI, the expressions of fibrosis-related proteins, such as hypoxia-inducible factor-1α, collagen I, and collagen III, are elevated in the corpus cavernosum [46]. Although fibrosis occurs to protect injured tissues, it often results in irreversible cellular and organ dysfunctions [47]. Therefore, it is crucial that nerve regeneration is induced in the early stage of the injury for the recovery of erectile functions. In the present study, we implanted the therapeutic cells – SCs or SKP-SCs – immediately after inducing BCNI and evaluated the penile erectile functions of the subjects and changes in their corpus cavernosum. The collagen-to-smooth muscle ratio, an indicator of intact smooth muscle content, was significantly higher in the SKP-SC and primary SC groups than in the PBS group. The result indicated that a significant level of fibrosis caused by BCNI could be prevented by both SKP-SCs and primary SCs in the penile corpus cavernosum. A consistent trend was observed in the expression of desmin, a marker of smooth muscle, in which both SKP-SC and primary SC groups showed higher expressions than the PBS group. The prevention of fibrosis is one of the most important mechanisms for the protection of erectile tissues. We then proceeded to evaluate the TGFβ-Smad2/3 signaling pathway to further investigate the mechanisms of fibrosis in the corpus cavernosum. In response to tissue damage, TGF-β1 strengthens and stimulates the phosphorylation of Smad2/3 [48], which is activated by serine/threonine phosphorylation and binds to the co-activator to form a complex. The formed complex subsequently moves into the nucleus and regulates the transcription of fibrosis-related target genes [49]. We used histochemical staining to quantify the changes in the expression of TGF-β1 and p-Smad2/3. The PBS group showed the highest levels of TGF-β1 and p-Smad2/3 among the treatment groups, indicating that severe fibrosis had occurred in the corpus cavernosum two weeks after BCNI induction. The SKP-SC and primary SC groups showed significantly lower levels of TGF-β1 and p-Smad2/3 than those in the PBS group. These results indicated that the TGFβ-Smad2/3 signaling pathway was significantly downregulated in the SKP-SC and primary SC groups to prevent the progression of fibrosis in the corpus cavernosum.

To date, a couple of stem cell application methods have been explored to treat ED in a BCNI rat model: (1) transplantation around MPG and (2) transplantation in the penis cavernous via intracavernous injection (ICI). In this study, SKP-SCs and primary SCs were transplanted around MPG as we rationally assumed that SKP-SCs exert their therapeutic effects mainly by promoting the regeneration of the MPG and CN. The transplantation of SKP-SCs around the MPG enabled the cells to secrete different cytokines directly to the injured nerves for repair functions. Furthermore, ICI of stem cells can cause various undesired complications, including pulmonary embolism caused by cell migration in circulation [50]. In contrast, transplantation around MPG poses virtually no risk of cells entering the circulation, and therefore the potential complications could be avoided in our therapeutic strategy.

## Limitations of the study

At the same time, our study still has some limitations. First, we measured the erectile function after the implantation of SKP-SCs and SCs, but the deeper mechanisms are still to be discovered. Second, the implantation of SKP-SCs and SCs was immediately after BCNI, whereas in the clinic, ED is often observed relatively long after surgery-related nerve injury. Hence, future studies should evaluate the effect of treatment at different time points after BCNI. A more rational experimental design is necessary to evaluate the role of SKP-SCs in the treatment of BCNI-induced ED.

## Conclusion

The present study demonstrated that SKP-SCs can effectively repair the injured cavernous nerve, protect the functional penile tissues, and delay or prevent fibrosis in the corpus cavernosum. The erectile function was restored as a consequence. Therefore, the application of SKP-SCs is a potentially effective method to restore erectile function in patients with nerve injury-related ED.

## Data Availability

The datasets used and/or analyzed during the current study are available from the corresponding author on reasonable request.

## Abbreviations

AOD: Average optical density
BCNI: Bilateral cavernous nerve injury
BDNF: Brain-derived neurotrophic factor
CNS: Central nerve system
Col VI: Collagen VI
DAPI: 4',6-Diamidino-2-phenylindole
DMEM: Dulbecco’s Modified Eagle’s Medium
ED: Erectile dysfunction
EGF: Epidermal growth factor
ELISA: Enzyme-linked immunosorbent assay
eNOS: Endothelial nitric oxide synthase
FGF: Fibroblast growth factor
GDNF: Glial cell-derived neurotrophic factor
GFAP: Glial fibrillary acidic protein
HBSS: Hank’s balanced salt solution
ICP: Intracavernous pressure
ICI: Intracavernous injection
MAP: Mean arterial blood pressure
MBP: Myelin basic protein
MCP-1: Monocyte chemotactic protein 1
mICP: Maximum intracavernous pressure
MPG: Major pelvic ganglion
NGF: Nerve growth factor
nNOS: Neuronal nitric oxide synthase
PAN: Pelvic autonomic nerve
PBS: Phosphate buffered saline
PDE5I: Phosphodiesterase type 5 inhibitor
PMA: Phorbol 12-myristate 13-acetate
RPMI-1640: Roswell Park Memorial Institute-1640
SC: Schwann cell
SD: Sprague–Dawley
SKP-SC: Skin-derived precursor Schwann cell
TGF-β1: Transforming growth factor-β1

## References

[1] Towe M, Huynh LM, El-Khatib F, Gonzalez J, Jenkins LC, Yafi FA (2019). A review of male and female sexual function following colorectal surgery. Sex Med Rev.

[2] Maas CP, Moriya Y, Steup WH, Klein Kranenbarg E, van de Velde CJ (2000). A prospective study on radical and nerve-preserving surgery for rectal cancer in the Netherlands. Eur J Surg Oncol.

[3] Celentano V, Fabbrocile G, Luglio G, Antonelli G, Tarquini R, Bucci L (2010). Prospective study of sexual dysfunction in men with rectal cancer: feasibility and results of nerve sparing surgery. Int J Colorectal Dis.

[4] Liu Z, Huang M, Kang L, Wang L, Lan P, Cui J (2016). Prognosis and postoperative genital function of function-preservative surgery of pelvic autonomic nerve preservation for male rectal cancer patients. BMC Surg.

[5] Bergman J, Gore JL, Penson DF, Kwan L, Litwin MS (2009). Erectile aid use by men treated for localized prostate cancer. J Urol.

[6] Pavlovich CP, Levinson AW, Su LM, Mettee LZ, Feng Z, Bivalacqua TJ (2013). Nightly vs on-demand sildenafil for penile rehabilitation after minimally invasive nerve-sparing radical prostatectomy: results of a randomized double-blind trial with placebo. BJU Int.

[7] Montorsi F, Brock G, Lee J, Shapiro J, Van Poppel H, Graefen M (2008). Effect of nightly versus on-demand vardenafil on recovery of erectile function in men following bilateral nerve-sparing radical prostatectomy. Eur Urol.

[8] Montorsi F, Brock G, Stolzenburg JU, Mulhall J, Moncada I, Patel HR (2014). Effects of tadalafil treatment on erectile function recovery following bilateral nerve-sparing radical prostatectomy: a randomised placebo-controlled study (REACTT). Eur Urol.

[9] Palma CA, Keast JR (2006). Structural effects and potential changes in growth factor signalling in penis-projecting autonomic neurons after axotomy. BMC Neurosci.

[10] Zhou F, Hui Y, Xin H, Xu YD, Lei HE, Yang BC (2017). Therapeutic effects of adipose-derived stem cells-based microtissues on erectile dysfunction in streptozotocin-induced diabetic rats. Asian J Androl.

[11] Yang M, Sun JY, Ying CC, Wang Y, Guo YL (2020). Adipose-derived stem cells modified by BDNF gene rescue erectile dysfunction after cavernous nerve injury. Neural Regen Res.

[12] Bunge RP (1994). The role of the Schwann cell in trophic support and regeneration. J Neurol.

[13] Fu SY, Gordon T (1997). The cellular and molecular basis of peripheral nerve regeneration. Mol Neurobiol.

[14] Tofaris GK, Patterson PH, Jessen KR, Mirsky R (2002). Denervated Schwann cells attract macrophages by secretion of leukemia inhibitory factor (LIF) and monocyte chemoattractant protein-1 in a process regulated by interleukin-6 and LIF. J Neurosci.

[15] Guest JD, Rao A, Olson L, Bunge MB, Bunge RP (1997). The ability of human Schwann cell grafts to promote regeneration in the transected nude rat spinal cord. Exp Neurol.

[16] Nishiura Y, Brandt J, Nilsson A, Kanje M, Dahlin LB (2004). Addition of cultured Schwann cells to tendon autografts and freeze-thawed muscle grafts improves peripheral nerve regeneration. Tissue Eng.

[17] Toma JG, Akhavan M, Fernandes KJ, Barnabé-Heider F, Sadikot A, Kaplan DR (2001). Isolation of multipotent adult stem cells from the dermis of mammalian skin. Nat Cell Biol.

[18] Toma JG, McKenzie IA, Bagli D, Miller FD (2005). Isolation and characterization of multipotent skin-derived precursors from human skin. Stem Cells.

[19] Biernaskie JA, McKenzie IA, Toma JG, Miller FD (2006). Isolation of skin-derived precursors (SKPs) and differentiation and enrichment of their Schwann cell progeny. Nat Protoc.

[20] McKenzie IA, Biernaskie J, Toma JG, Midha R, Miller FD (2006). Skin-derived precursors generate myelinating Schwann cells for the injured and dysmyelinated nervous system. J Neurosci.

[21] Biernaskie J, Sparling JS, Liu J, Shannon CP, Plemel JR, Xie Y (2007). Skin-derived precursors generate myelinating Schwann cells that promote remyelination and functional recovery after contusion spinal cord injury. J Neurosci.

[22] May Z, Kumar R, Fuehrmann T, Tam R, Vulic K, Forero J (2018). Adult skin-derived precursor Schwann cell grafts form growths in the injured spinal cord of Fischer rats. Biomed Mater.

[23] Fang JF, Jia CC, Zheng ZH, Ye XL, Wei B, Huang LJ (2016). Periprostatic implantation of neural differentiated mesenchymal stem cells restores cavernous nerve injury-mediated erectile dysfunction. Am J Transl Res.

[24] You D, Jang MJ, Lee J, Jeong IG, Kim HS, Moon KH (2013). Periprostatic implantation of human bone marrow-derived mesenchymal stem cells potentiates recovery of erectile function by intracavernosal injection in a rat model of cavernous nerve injury. Urology.

[25] Zhu Z, Ding J, Ma Z, Iwashina T, Tredget EE (2017). Alternatively activated macrophages derived from THP-1 cells promote the fibrogenic activities of human dermal fibroblasts. Wound Repair Regen.

[26] Katebi S, Esmaeili A, Ghaedi K, Zarrabi A (2019). Superparamagnetic iron oxide nanoparticles combined with NGF and quercetin promote neuronal branching morphogenesis of PC12 cells. Int J Nanomedicine.

[27] Kim JA, Lee N, Kim BH, Rhee WJ, Yoon S, Hyeon T (2011). Enhancement of neurite outgrowth in PC12 cells by iron oxide nanoparticles. Biomaterials.

[28] Lin G, Li H, Zhang X, Wang J, Zaid U, Sanford MT (2015). Novel therapeutic approach for neurogenic erectile dysfunction: effect of neurotrophic tyrosine kinase receptor type 1 monoclonal antibody. Eur Urol.

[29] Rutkowski JL, Tennekoon GI, McGillicuddy JE (1992). Selective culture of mitotically active human Schwann cells from adult sural nerves. Ann Neurol.

[30] Rutkowski JL, Kirk CJ, Lerner MA, Tennekoon GI (1995). Purification and expansion of human Schwann cells in vitro. Nat Med.

[31] Fernandes KJ, McKenzie IA, Mill P, Smith KM, Akhavan M, Barnabé-Heider F (2004). A dermal niche for multipotent adult skin-derived precursor cells. Nat Cell Biol.

[32] Khuong HT, Kumar R, Senjaya F, Grochmal J, Ivanovic A, Shakhbazau A (2014). Skin derived precursor Schwann cells improve behavioral recovery for acute and delayed nerve repair. Exp Neurol.

[33] Cho MC, Park K, Kim SW, Paick JS (2015). Restoration of erectile function by suppression of corporal apoptosis, fibrosis and corporal veno-occlusive dysfunction with rho-kinase inhibitors in a rat model of cavernous nerve injury. J Urol.

[34] Hatzimouratidis K, Burnett AL, Hatzichristou D, McCullough AR, Montorsi F, Mulhall JP (2009). Phosphodiesterase type 5 inhibitors in postprostatectomy erectile dysfunction: a critical analysis of the basic science rationale and clinical application. Eur Urol.

[35] Boyd JG, Gordon T (2003). Glial cell line-derived neurotrophic factor and brain-derived neurotrophic factor sustain the axonal regeneration of chronically axotomized motoneurons in vivo. Exp Neurol.

[36] Stratton JA, Shah PT, Kumar R, Stykel MG, Shapira Y, Grochmal J (2016). The immunomodulatory properties of adult skin-derived precursor Schwann cells: implications for peripheral nerve injury therapy. Eur J Neurosci.

[37] Van Steenwinckel J, Auvynet C, Sapienza A, Reaux-Le Goazigo A, Combadière C (2015). Stromal cell-derived CCL2 drives neuropathic pain states through myeloid cell infiltration in injured nerve. Brain Behav Immun.

[38] Namikawa K, Okamoto T, Suzuki A, Konishi H, Kiyama H (2006). Pancreatitis-associated protein-III is a novel macrophage chemoattractant implicated in nerve regeneration. J Neurosci.

[39] Perrin FE, Lacroix S, Avilés-Trigueros M, David S (2005). Involvement of monocyte chemoattractant protein-1, macrophage inflammatory protein-1alpha and interleukin-1beta in Wallerian degeneration. Brain.

[40] Chen P, Cescon M, Zuccolotto G, Nobbio L, Colombelli C, Filaferro M (2015). Collagen VI regulates peripheral nerve regeneration by modulating macrophage recruitment and polarization. Acta Neuropathol.

[41] Mokarram N, Merchant A, Mukhatyar V, Patel G, Bellamkonda RV (2012). Effect of modulating macrophage phenotype on peripheral nerve repair. Biomaterials.

[42] Quarta S, Baeumer BE, Scherbakov N, Andratsch M, Rose-John S, Dechant G (2014). Peripheral nerve regeneration and NGF-dependent neurite outgrowth of adult sensory neurons converge on STAT3 phosphorylation downstream of neuropoietic cytokine receptor gp130. J Neurosci.

[43] Giuliano F, Rampin O (2004). Neural control of erection. Physiol Behav.

[44] Chen YL, Chao TT, Wu YN, Chen MC, Lin YH, Liao CH (2018). nNOS-positive minorbranches of the dorsal penile nerves is associated with erectile function in the bilateral cavernous injury model of rats. Sci Rep.

[45] Song SH, Park K, Kim SW, Paick JS, Cho MC (2015). Involvement of Rho-Kinase/LIM Kinase/Cofilin Signaling Pathway in Corporal Fibrosis after Cavernous Nerve Injury in Male Rats. J Sex Med.

[46] Leungwattanakij S, Bivalacqua TJ, Usta MF, Yang DY, Hyun JS, Champion HC (2003). Cavernous neurotomy causes hypoxia and fibrosis in rat corpus cavernosum. J Androl.

[47] Campana L, Iredale JP (2017). Regression of Liver Fibrosis. Semin Liver Dis.

[48] Okamoto Y, Hasegawa M, Matsushita T, Hamaguchi Y, Huu DL, Iwakura Y (2012). Potential roles of interleukin-17A in the development of skin fibrosis in mice. Arthritis Rheum.

[49] Zhang Z, Xue Z, Liu Y, Liu H, Guo X, Li Y (2018). MicroRNA-181c promotes Th17 cell differentiation and mediates experimental autoimmune encephalomyelitis. Brain Behav Immun.

[50] Xu Y, Yang Y, Zheng H, Huang C, Zhu X, Zhu Y (2020). Intracavernous injection of size-specific stem cell spheroids for neurogenic erectile dysfunction: Efficacy and risk versus single cells. EBioMedicine.

